# Anthelmintic Potential and In Silico Studies of Ricinoleic Acid from the Seed Oil of *Ricinus communis* L.

**DOI:** 10.3390/ijms26041636

**Published:** 2025-02-14

**Authors:** Temesgen Berhanu, Eyael Tewelde, Mariamawit Y. Yeshak, Daniel Bisrat, Kaleab Asres

**Affiliations:** 1 Department of Pharmaceutical Chemistry and Pharmacognosy, School of Pharmacy, College of Health Sciences, Addis Ababa University, Addis Ababa P.O. Box 1176, Ethiopia; tederastame@gmail.com (T.B.); eyaeltd@gmail.com (E.T.); mariamawit.yonathan@aau.edu.et (M.Y.Y.); daniel.bisrat@aau.edu.et (D.B.); 2 Department of Pharmacognosy, School of Pharmacy, Dilla University, Dilla P.O. Box 419, Ethiopia

**Keywords:** anthelmintic, *Ricinus communis*, fatty acids, *Caenorhabditis elegans*, ricinoleic acid, succinate dehydrogenase (SDH), molecular docking, absorption, distribution, metabolism, and excretion (ADME)

## Abstract

The prevalence of human intestinal helminth parasitic infections is extensive, with over half of the global population estimated to suffer from these infections. Traditionally, various plant species, including *Ricinus communis* L. (Euphorbiaceae), are used to treat helminth infections. In this study, ricinoleic acid was isolated from the base hydrolysate of the petroleum ether seed extract of *R. communis* using column chromatography and transformed into ricinoleic acid methyl ester through esterification. The extract, ricinoleic acid and its methyl ester were evaluated for their anthelmintic activities against the model organism *Caenorhabditis elegans*. The results revealed that at a concentration of 1 mg/mL, ricinoleic acid and its methyl ester killed 97.40% and 97.83% of *C. elegans* worms, respectively. Molecular docking studies of ricinoleic acid on succinate dehydrogenase (SDH), glucose-6-phosphate 1-dehydrogenase (G6PD), and tubulin beta-2 chain (TBB2C) revealed that ricinoleic acid has a more favorable interaction with succinate dehydrogenase (−5.408 kcal/mol) compared to glucose-6-phosphate 1-dehydrogenase (−3.758 kcal/mol) and tubulin beta-2 chain (−1.444 kcal/mol). Furthermore, Absorption, Distribution, Metabolism, and Excretion (ADME) analyses unveiled that ricinoleic acid adheres to Lipinski’s rule of five, positioning it as a potential compound to treat helminths. The current study demonstrated that *R. communis* seed oil possesses genuine anthelmintic activity against *C. elegans*, which is likely due to ricinoleic acid.

## 1. Introduction

Helminths have been a problem for humans and animals throughout history [1]. Helminthic infections are highly prevalent, affecting more than half of the world’s population, with children and pregnant women being particularly vulnerable [2]. These infections also have a significant impact on animal health, leading to substantial economic losses and disability-adjusted life years [3,4,5].

There is a concern regarding the emergence of drug-resistant worms due to the limited number of medications available to treat helminth infections. When parasites are exposed to the same drug repeatedly, those that survive may pass on genetic traits that make their offspring more resistant to the anthelmintic drug [6]. Therefore, there is a need to explore new drugs derived from natural sources to combat these parasitic worms. Traditional medicine, with its indigenous knowledge, could be a valuable resource for finding anthelmintics, as it identifies which plants are effective against specific diseases. Plant-based anthelmintic medications have been reported to have fewer side effects than synthetic drugs [7,8,9]. Drugs against helminths act by mechanisms such as binding to nicotinic cholinergic receptors, inhibiting acetylcholinesterase, increasing calcium permeability, binding to β-tubulin, and inhibiting oxidative phosphorylation and arachidonic acid metabolism [10].

The use of natural products from plants, animals, and marine organisms as medicines to treat and alleviate illnesses dates back thousands of years. Fossil records suggest that using natural products as medicine by humans can be traced back to at least 60,000 years [11]. Even today, approximately 60–80% of the world’s population relies on traditional medicines for treating common illnesses [12,13]. In many indigenous communities, plants play a central role in combating various diseases in humans and livestock. Medicinal plants are a valuable source of ingredients that can be utilized in drug development and synthesis [14]. Various plants from different families are used worldwide to treat helminthiasis. Local communities in Ethiopia use plants such as *Agati gratifola*, *Agrimonia eupatori*, *Butea fondosa*, *Carica papaya*, *Combretum mucoreatum*, *Cucurbita moschata*, *Hagenia abyssinica*, *Helleborus niger,* and *Mangifera indica* to treat helminthic infections in humans and livestock [15,16,17].

*Ricinus communis* L., commonly known as the castor plant, belongs to the spurge family, Euphorbiaceae, which comprises approximately 6745 species in 218 genera [18,19]. The castor plant has been cultivated since 4000 A.D. and has numerous uses, with every part of the plant being utilized [20,21]. Previous studies have demonstrated that *R. communis* leaves [22], bark [23], and seed oil [24] possess genuine anthelmintic activity against the adult earthworm *Pheretima posthuma*. This study investigates the in vitro anthelmintic effects of castor oil, its primary hydrolyzed fatty acid (ricinoleic acid), and ricinoleic acid methyl ester against the nematode *Caenorhabditis elegans*. *C. elegans* is a free-living, transparent roundworm approximately 1 mm in length, commonly found in soil. It is widely used as a model organism in molecular and developmental biology due to its simplicity and well-characterized genome [25]. A docking study of ricinoleic acid on SDH (succinate dehydrogenase), G6PD (glucose-6-phosphate 1-dehydrogenase), and TBB2C (tubulin beta-2 chain) was also carried out to get further insight into the possible mechanism of the fatty acid.

## 2. Results

### 2.1. Extraction Yield

In this study, the powdered dried seeds of *R. communis* were subjected to cold maceration in petroleum ether, affording a yellow-colored oil. The percentage yield of the oil was 8.4% (*w*/*w*, based on the dried weight). Previous studies indicate the yield of castor oil from castor beans is between 34.6 and 56.6% [26]. The lower oil yield obtained in this study could be attributed to variations caused by the variety of the plant, climatic conditions, and geographical location where the seeds were collected.

### 2.2. In Vitro Anthelmintic Activity

**Figure 1:**
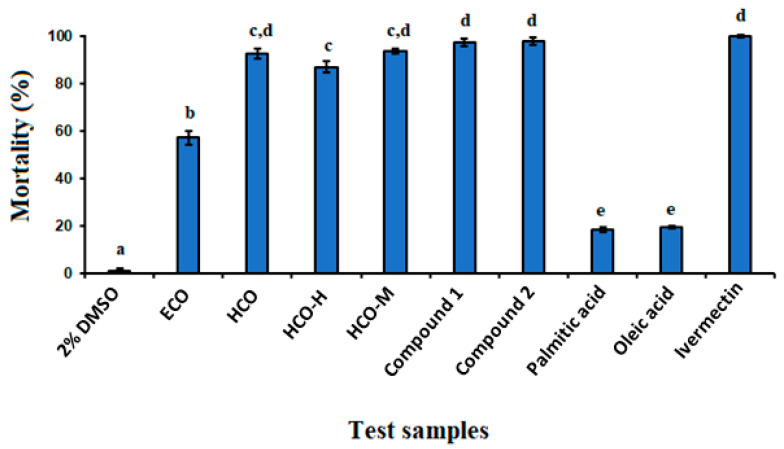
Anthelmintic activities of test samples against *Caenorhabditis elegans* at a concentration of 1 mg/mL (2% DMSO: negative control; ECO: fixed oil extracted from the seeds of *Ricinus communis*; HCO: hydrolysate of ECO; HCO-H, hexane fraction from the hydrolysate HCO-M: methanol fraction from the hydrolysate; Compound **1**: ricinoleic acid isolated from *Ricinus communis*; Compound **2**: ricinoleic acid methyl ester; palmitic acid; oleic acid; Ivermectin (10 μg/mL): positive control. Note: Different letters above the graph bars represent statistical differences between the groups (*p* < 0.05; one-way ANOVA, Tukey’s multiple comparison test).

The oil isolated from *R. communis* seeds showed significant anthelmintic activity (*p* < 0.05) against *C. elegans* worms. A 1 mg/mL concentration resulted in a mortality rate of 57.20%, compared to the negative control (2% DMSO) (Figure 1). Previous studies proved that fixed oils derived from various plants exhibit anthelmintic activity. For instance, *Cucurbita pepo* seed oil showed 89% mortality against *Heligmosoides bakeri* [27]. The anthelmintic properties of several fixed oils have been attributed to the presence of medium- and long-chain fatty acids [27,28]. For instance, the fixed oil from *Jatropha curcas* seeds, which contain palmitic acid as a major constituent (55%), showed significant anthelmintic activity [28]. Thus, the anthelmintic activity of castor oil is believed to be attributed to the presence of free fatty acids in the oil, as observed in other studies [29]. Consequently, the oil obtained from the seeds of *R. communis* was further hydrolyzed to obtain free fatty acids.

The hydrolysate obtained from *R. communis* oil displayed significant anthelmintic activity (*p* < 0.05) at a concentration of 1 mg/mL, resulting in the death of 92.37% of *C. elegans* worms compared to the negative control (Figure 1). The hydrolysate was then partitioned between *n*-hexane and methanol to isolate active compounds for further anthelmintic activity testing. While both fractions exhibited anthelmintic activity, the methanol fraction showed stronger activity. At a concentration of 1 mg/mL, the *n*-hexane and methanol fractions caused 86.93% and 93.60% mortality of *C. elegans* worms, respectively (Figure 1). The most active methanol fraction of the hydrolysate was subjected to column chromatography over silica gel, leading to the isolation of compound **1**. Compound **1** was further esterified to obtain its methyl ester, compound **2**.

### 2.3. Structural Elucidation of Compounds ***1*** and ***2***

**Figure 2:**
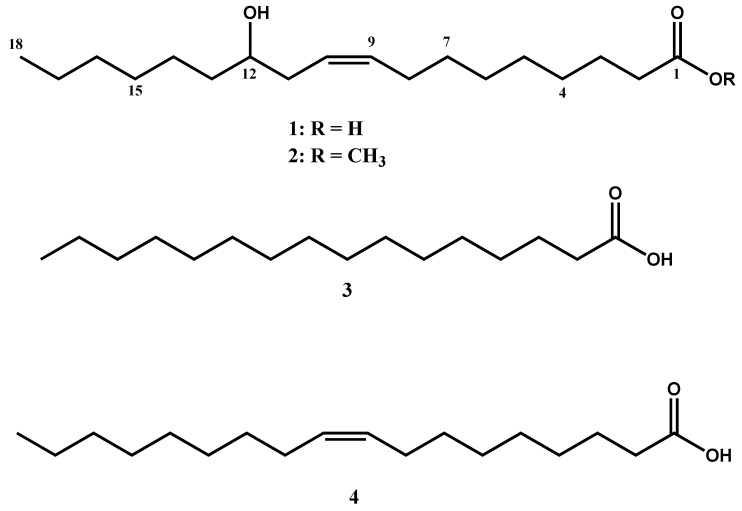
The structural formulas of ricinoleic acid (**1**) isolated from *Ricinus communis*, its methyl ester derivative (**2**), palmitic acid (**3**), and oleic acid (**4**).

Compound **1** was isolated as a white waxy solid from acetone, with an *R_f_* value of 0.6 in the *n*-hexane/ethyl acetate (4:1) solvent system. Compound **1** gave a pseudomolecular ion at *m/z* = 297.4 [M-H]^−^ in the negative mode ESI-MS, indicating a relative molecular mass of 298 mu. This, along with the ^1^H and ^13^C NMR spectral data (see Materials and Methods section), characterized compound **1** as 9Z-12-hydroxyoctadec-9-enoic acid, commonly known as ricinoleic acid (Figure 2). Previous studies have also identified ricinoleic acid from the seeds, roots, and leaves of *R. communis* [30,31], as well as from the seed oil of *Phyllantus niruri* [32].

Compound **2** displayed similar signal patterns in the ^1^H, ^13^C, and DEPT-NMR spectra to that of ricinoleic acid (**1**), except for an additional signal due to the presence of a methoxy group moiety (OCH_3_; 3.67 ppm in ^1^H and 51.5 ppm in ^13^C-NMR). Based on the ^1^H and ^13^C spectral data (see Materials and Methods section), compound **2** was identified as ricinoleic acid methyl ester (Figure 2).

### 2.4. Anthelmintic Activity of Ricinoleic Acid and Methyl Ricinoleate

Ricinoleic acid exhibited strong anthelmintic activity, causing 97.40% mortality of *C. elegans* worms at a concentration of 1 mg/mL. Similarly, ricinoleic acid methyl ester killed 97.83% of the worms at the same concentration, indicating that esterification does not affect the anthelmintic activity of the free fatty acid. It is interesting to note that the anthelmintic activity of ricinoleic acid was significantly higher than that of palmitic (**3**) acid and oleic acid (**4**), which caused mortality of only 18.81% and 19.83%, respectively, at the same concentration (Figure 1). This implies that the hydroxyl group of ricinoleic acid is crucial in enhancing the anthelmintic activity of the compound.

### 2.5. Molecular Docking Analysis

#### 2.5.1. Homology Modeling and Structure Validations of Modeled Proteins

**Figure 3:**
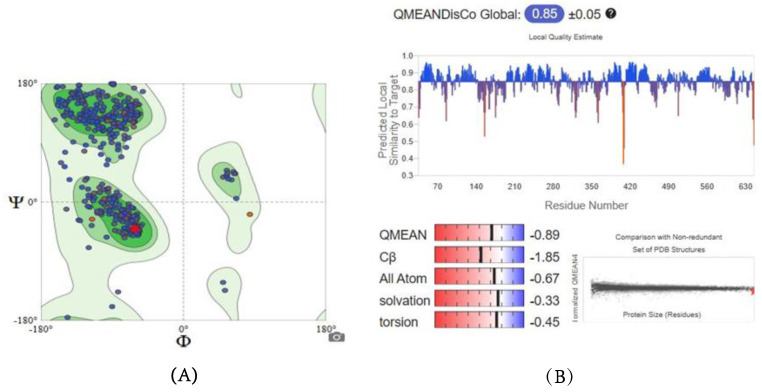
Ramachandran Plot (**A**) and QMEANDisCo (**B**) of homology modeled SDH structure.

Since the experimental three-dimensional structures for SDH, G6PD, and TBB2C were not available, a homology modelling approach was used to predict their structures using the Swiss-Model approach. The quality of the obtained models was assessed by examining parameters such as Global Model Quality Estimation (GMQE), Qualitative Model Energy Analysis (QMEAN), and Ramachandran Plots, and the results are outlined in Table 1. Figure 3 depicts the graphical representation of GMQE, QMEAN, and Ramachandran Plots of homology modeled SDH structure.

The 3D structure of SDH was developed using the Swiss-Model, with a QMEAN score of −0.89 and a GMQE score of 0.86. These scores are considered reliable, as a QMEAN score below −4.0 indicates low structural quality, and GMQE scores range from 0 to 1, with higher scores reflecting greater reliability. Additionally, the predicted SDH model exhibits a favorable outcome of 96.39%, comfortably falling within the accepted range. These results suggest that the newly predicted models are both reliable and closely approximate native conditions.

**Figure 4:**
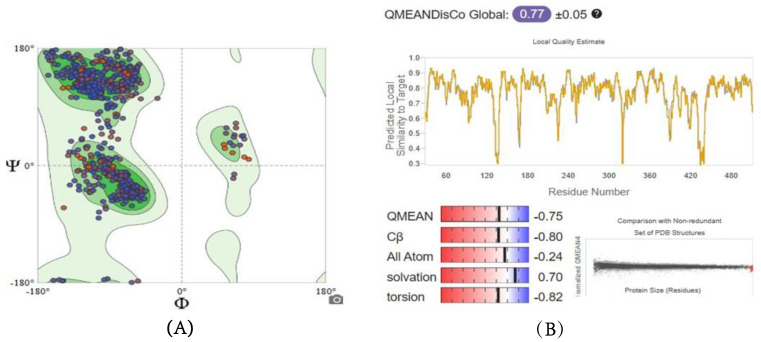
Ramachandran Plot (**A**) and QMEANDisCo (**B**) of homology modeled G6PD structure.

**Figure 5:**
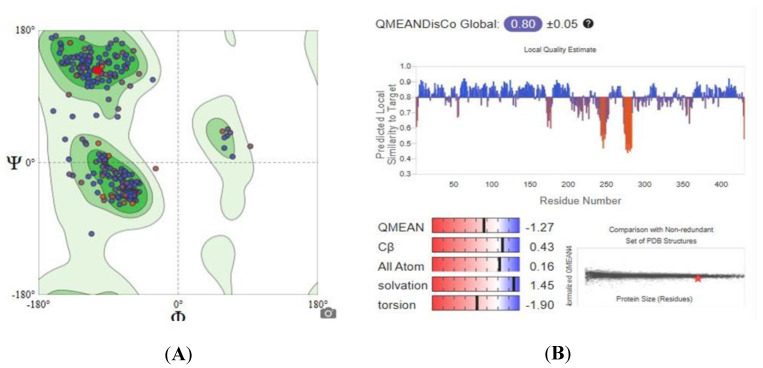
Ramachandran Plot (**A**) and QMEANDisCo (**B**) of homology modeled TBB2 structure.

Similarly, modeled structures for 6GPD and TBB2C were generated, and both exhibited acceptable quality parameters. In Figure 4 and Figure 5, the GMQE, QMEAN, and Ramachandran Plots for G6PD and TBB2C protein models are represented, respectively. Consequently, it is deemed that the models are suitable for the objectives of this study.

#### 2.5.2. Docking Results

**Figure 6:**
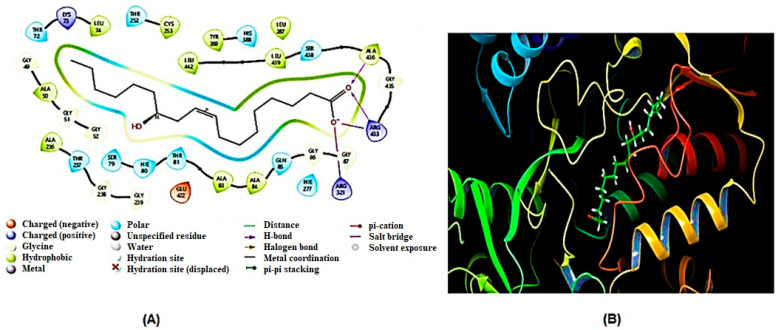
The (**A**) 2D and (**B**) 3D binding interaction of ricinoleic acid with SDH.

**Figure 7:**
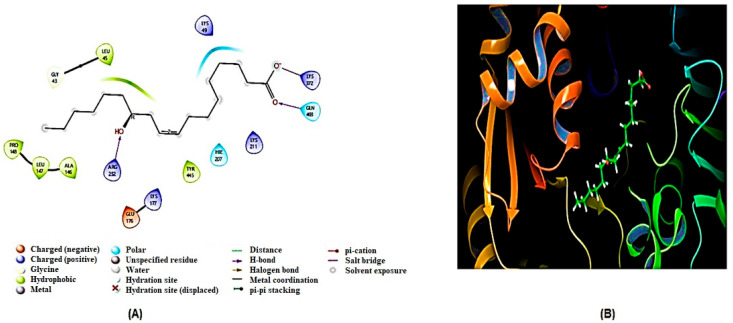
The (**A**) 2D and (**B**) 3D binding interaction of ricinoleic acid with G6PD.

**Figure 8:**
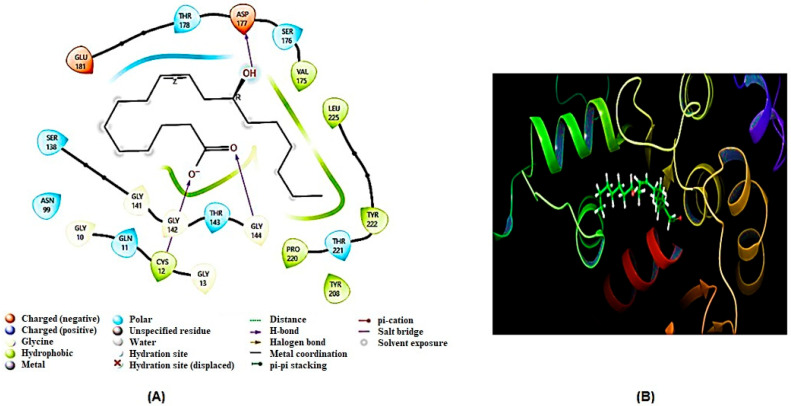
The (**A**) 2D and (**B**) 3D binding interaction of ricinoleic acid with TBB2.

After generating homology models, molecular docking analysis of ricinoleic acid was conducted on three key homology-modeled proteins—SDH, G6PD, and TBB2C from *C. elegans*—identified as potential targets for anthelmintic drugs. The comparison was made with the reference compound mebendazole, a well-known anthelmintic drug that shows excellent binding affinity to some of the aforementioned molecular targets [33,34]. The results of the molecular docking are summarized in Table 2 and Table 3, and visual representations of the ligand–receptor interacting complex are depicted in Figure 6, Figure 7 and Figure 8.

The present findings indicated a strong interaction between SDH and ricinoleic acid, exceeding the interactions observed with the other two examined enzymes (G6PD and TBBC), as evidenced by their respective docking values (Table 2). SDH exhibited the most favorable interaction with a docking score of −5.408 kcal/mol, while the reference compound mebendazole showed a docking score of −6.24 kcal/mol. The presence of flavin adenine dinucleotide (FAD) coenzyme in the SDH enzyme is pivotal for its interaction with ricinoleic acid. Notably, ricinoleic acid was found to bind to the FAD binding site in SDH through the formation of three hydrogen bonds with specific amino acid residues (Arg433, Ala436, Ala236). Additionally, Table 3 indicates the involvement of several other amino acid residues in this interaction.

Ricinoleic acid exhibited a moderate binding affinity with 6GPD at the substrate-binding site, as evidenced by a docking score of −3.758 kcal/mol (Table 2). The interaction involved both hydrogen bonds and non-hydrogen bond interactions with amino acids of the 6GPD enzyme, as detailed in Table 3. However, it is noteworthy that ricinoleic acid did not demonstrate a significant docking score at the binding sites of TBB2C (−1.444 kcal/mol).

#### 2.5.3. ADME (Absorption, Distribution, Metabolism, and Excretion) Property Prediction

The QikProp module employs Lipinski’s rule of five to predict the behavior of ricinoleic acid within the biological system [35]. This provides valuable insights into the pharmacokinetic profile of ricinoleic acid, as summarized in Table 4. The ADME analysis of ricinoleic acid has not violated any of the criteria for Lipinski’s rule of five [36], positioning it as a potential compound for treating helminths.

## 3. Discussion

Helminthic infections are highly prevalent, affecting more than half of the world’s population, with children and pregnant women being particularly vulnerable [2]. Limited resources in developing nations hinder anthelmintic drug development, despite a high prevalence of parasitic worms. Pharmaceutical companies show little interest due to challenges in establishing a profitable market for these drugs [37]. Therefore, the search for new, safe, and affordable anthelmintic drugs is crucial.

In the current study, the free-living nematode *C. elegans* was used as a model organism to evaluate the anthelmintic activity of *R. communis* seed oil. *C. elegans* is a good model organism due to its ease of laboratory maintenance and its similarities to the parasitic worms [38]. For example, it was scientifically proved that the animal parasitic suborder Strongylida, which includes the roundworms *Ancylostoma duodenale* and *Necator americanus*, the pathogens that cause hookworm infections in humans and other mammals, are closely related to *C. elegans* [39].

*R. communis* seeds are traditionally used in folk medicine for treating various ailments, including worm infections. In this study, the oil isolated from *R. communis* seeds demonstrated significant anthelmintic activity against *C. elegans* worms, resulting in a mortality rate of 57.20% at a concentration of 1 mg/mL. Ricinoleic acid, which constitutes about 90% of the total fatty acids in the seed oil, obtained through acid hydrolysis, exhibited a much stronger anthelmintic activity, causing 97.40% mortality of *C. elegans* worms at the same concentration. Similarly, ricinoleic acid methyl ester, derived by esterification of ricinoleic acid, induced 97.83% mortality at 1 mg/mL, indicating that esterification does not significantly affect the anthelmintic activity of the free fatty acid. It is interesting to note that the anthelmintic activity of ricinoleic acid was significantly higher than those demonstrated by palmitic acid (**3**) and oleic acid (**4**), which caused mortality of only 18.81% and 19.83%, respectively, at a concentration of 1 mg/mL (Figure 1). This implies that the hydroxyl group of ricinoleic acid is crucial in enhancing the anthelmintic activity of the compound. Previous studies have also shown that the presence of oxygen at position 12 in unsaturated fatty acids, such as 12 hydroxyoleic acid, hydroxyelaidic acid, 12-oxo-octadecenoic acid, and vernolic acid methyl ester, significantly enhances anthelmintic activity against *C. elegans* [40].

Stadler et al. [41] demonstrated that S-coriolic acid ((9Z,11E,13S)-13-hydroxyoctadeca-9,11-dienoic acid) isolated from the submerged cultures of the mushroom *Pleurotus pulmonarius* possesses strong anthelmintic activity against *C. elegans.* It appears that unsaturation and hydroxylation increase the activity of fatty acids against this model organism. A study conducted by Hirazawa et al. [42] showed that medium-chain fatty acids (carbon numbers C_6_–C_10_) possess good activity against the monogenean worm *Heterobothrium okamotoi*. This finding was significant because *H. okamotoi* is highly pathogenic and resistant to chemical treatments. Other studies have also demonstrated that medium- and long-chain fatty acids inhibit the growth of *C. elegans* and parasitic worms [29,42]. Several fatty acids, including caprylic acid, stearidonic acid, eicosapentaenoic acid, alpha-linolenic acid, docosahexaenoic acid, arachidonic acid, and pelargonic acid, displayed notable anthelmintic activity against various helminths [43,44].

It is suggested that fatty acids may alter the composition of helminths’ phospholipid membranes, leading to changes in fluidity and functionality, ultimately resulting in mortality [45,46]. Nematodes, including *C. elegans*, heavily rely on lipids for energy storage and metabolism [47]. As fatty acids are also described to be fundamental in *C. elegans* [48], other possible mechanisms of action for fatty acids, such as tegumental damages, cuticular distortion, and interaction with plasma membrane lipoproteins, have been proposed [47,49]. Therefore, the disruption of lipid homeostasis or interference with the nematode’s cuticle or hypodermis are potential mechanisms that contribute to the anthelmintic activity of fatty acids [45,50,51].

Fatty acids have been reported to possess a wide range of pharmacological activities, including anthelmintic action [45,52], which makes them a potential candidate to be explored as novel SDH, 6GPD, and TBB2C inhibitors. Previous studies have demonstrated that *R. communis* leaves [22], bark [23], and seed oil [24] possess genuine anthelmintic activity against the adult earthworm *Pheretima posthuma*.

Anthelmintic drugs act by targeting specific physiological or biochemical processes in helminths, causing paralysis, death, or expulsion from the host. The mode of action varies by drug class and helminth type [10]. One key target is the enzyme SDH, essential in the tricarboxylic acid (TCA) cycle, where it catalyzes the conversion of succinate to fumarate. In this study, ricinoleic acid demonstrated a strong interaction with SDH binding energy (−5.408 kcal/mol), disrupting the parasite’s energy production and leading to its death [53]. To our knowledge, this is the first report of a fatty acid interacting with SDH. While existing drugs less commonly utilize SDH for anthelmintic activity compared to those targeting neuromuscular systems (e.g., nicotinic acetylcholine receptors) or microtubule assembly, their mechanism of action is crucial for disrupting the parasite’s energy metabolism. These findings highlight ricinoleic acid’s potential as a promising candidate for anthelmintic drug development by targeting the metabolic pathways of helminths [53].

## 4. Materials and Methods

### 4.1. Plant Material

The seeds of *R. communis* were collected in April 2022 from Arsi Negelle town, west Arsi zone, Oromia regional state, 225 Km south of Addis Ababa in Ethiopia’s Central Rift Valley. The authenticity of the plant material was confirmed by Mr. Melaku Wondafrash, the National Herbarium, Department of Plant Biology and Biodiversity Management, College of Natural Sciences, Addis Ababa University (AAU), where a botanical specimen was deposited (collection number TB-002) for future reference.

### 4.2. Chemicals and Reagents

Petroleum ether, *n*-hexane, chloroform, ethyl acetate, acetone, hydrochloric acid, sulfuric acid (LOBA-Chemie, Mumbai, India), methanol (Sheba Pharmaceutical PLC, Addis Ababa, Ethiopia), palmitic acid, oleic acid (Amman Pharmaceutical Industries Co., Amman, Jordan); potassium hydroxide pellet, silica gel 60G/F254 (Carl Roth^®^, Karlsruhe, Germany); precoated analytical TLC, nematode growth media (NGM), Luria-Bertani (LB) media and agar, M9 buffer (Leibniz Institute of Plant Biochemistry (IPB) laboratory, Halle, Germany); dimethyl sulfoxide (DMSO) (Duchefa Biochemie, Haarlem, The Netherlands); penicillin–streptomycin solution (Capricorn Scientific GmbH, Ebsdorfergrund, Germany) were used as received. Ivermectin (Sigma-Aldrich, Sigma-Aldrich Chemie GmbH, Schnelldorf, Germany) was donated by the Ethiopian Pharmaceutical Manufacturing Factory (EPHARM), Addis Ababa, Ethiopia).

### 4.3. Test Organisms

The Bristol N2 wild-type strain of *Caenorhabditis elegans* was used for the anthelmintic assay. The worms were obtained from the Leibniz Institute of Plant Biochemistry (IPB) laboratory, Halle, Germany. The parasites were subsequently maintained at 20 °C on Nematode Growth Medium (NGM) agar, which was aseptically poured into 60 mm diameter Petri plates seeded with OP50 bacteria (*Escherichia coli* strain).

### 4.4. Extraction

The seeds of *R. communis* were cleaned, washed, dried under shade, and pulverized with a grinder. A portion of the dried seed powder (250 g) was macerated in petroleum ether in (1.5 L) with occasional shaking. The mixture was filtered using muslin cloth followed by Whatman No. 1 filter paper. To maximize the yield, the marc was re-macerated twice for 72 h. Then, the combined filtrates were evaporated under reduced pressure at a temperature not exceeding 40 °C in a rotary evaporator (Buchi Rota Vapor, R-200, Switzerland) to obtain the oil. The oil was then transferred to amber-colored glass vials and stored in a refrigerator at 4 °C for future use.

### 4.5. Hydrolysis of the Oil

The oil (20 g) was hydrolyzed by refluxing with 12% ethanolic potassium hydroxide solution (25 mL) for 1 h on a hot plate. After the organic solvent was evaporated, the resulting mixture was dissolved in deionized water (300 mL) and acidified with concentrated HCl until the pH = 1. The aqueous solution was partitioned with two 300 mL portions of ethyl acetate, and the organic solvent was removed using a rotary evaporator at 40 °C [54].

### 4.6. Isolation of Compound

The hydrolysate was partitioned between *n*-hexane (100 mL × 3) and methanol (100 mL × 3) to prepare *n*-hexane and methanol fractions. The methanol fraction was adsorbed on silica gel and chromatographed over a silica gel column using *n*-hexane and *n*-hexane-EtOAc gradients as eluent systems. Five 100 mL fractions were collected as follows: (F1: 100% *n*-hexane), (F2: *n*-hexane-EtOAc (90:10), (F3: *n*-hexane-EtOAc (80:20), (F4: *n*-hexane-EtOAc (70:30), (F5: *n*-hexane-EtOAc (60:40). The fractions were evaporated to dryness under reduced pressure using a rotary evaporator maintained at 40 °C. F3, which showed a reddish brown color on TLC, was taken in dry acetone and kept in a refrigerator overnight. After 24 h, the sample was taken out of the refrigerator and decanted. The resulting white amorphous solid substance (compound **1**) was stored in a refrigerator until use.

### 4.7. Esterification of Compound ***1***

Potassium hydroxide (5%) in anhydrous methanol (10 mL) was added to a solution of compound **1** (50 mg) in *n*-hexane (5 mL). The reaction mixture was heated at 50 °C for 15 min. After completion of the reaction, a few drops of glacial acetic acid were added, followed by the addition of 50 mL of water and 50 mL of *n*-hexane. The solution was shaken vigorously in a separatory funnel, and the upper organic layer was taken and dried. The dried sample designated compound **2** was then analyzed by TLC [55].

Compound **1**: White amorphous solid substance; R*_f_* value of 0.6 (*n*-hexane: ethyl acetate; 4:1); -ve ESI-MS (Figure S1): *m/z* = 297.4 [M-H]^−^, corresponding to a molecular formula of C_18_H_34_O_3_ (relative molecular mass of 298 mu [M-H]^−^); ^13^C-NMR (Figure S2): 14.4 (C-18); 22.7 (C-17); 24.9 (C-3); 25.7 (C-14); 27.1 (C-8); 29.0 (C-4); 29.1 (C-5); 29.2 (C-15); 29.4 (C-6); 29.5 (C-7); 31.7 (C-16); 34.1 (C-2); 35.6 (C-11); 36.9 (C-13); 70.2 (C-12); 128.2 (C-10); 130.1 (C-9); 174.9 (C-1); ^1^H-NMR (Figure S3): 0.86 (H-18, *t*, 3H); 1.16 (H-5, *m*, 2H); 1.24 (H-4,6,7,14-17, overlapping, 14H); 1.34 (H-13, *m*, 2H); 1.48 (H-3, *m*, 2H); 2.01 (H-11, *m*, 1H); 2.03 (H-11, *m*, 1H); 2.18 (H-2, *t*, 2H); 3.33 (H-12, *m*, 1H); 5.33 (H-10, *m*, 1H); 5.38 (H-9, *m*, 1H); 11.96 (OH, *s*, 1H). ^1^H and ^13^C-NMR spectral data of compound **1** were in good agreement with (9Z)-12-hydroxyoctadec-9-enoic acid (ricinoleic acid) spectral data reported in the National Center for Biotechnology Information (NCBI) [56].

Compound **2**: ^13^C-NMR (Figure S4) and DEPT-135 (Figure S5): 14.1 (C-18); 22.6 (C-17); 23.0 (C-3); 24.9 (C-14); 25.7 (C-8); 27.4 (C-4); 29.1 (C-5 & C-15); 29.4 (C-6); 29.6 (C-7); 31.9 (C-16); 34.1 (C-2); 35.3 (C-13); 36.8 (C-11); 51.5 (OCH_3_); 71.5 (C-12); 125.2 (C-10); 133.4 (C-9); 174.4 (C-1); ^1^H-NMR (Figure S6): 0.89 (H-18, *t*, 3H); 1.30 (H-15,16,14,4,5,6,7, Overlapping, 14H); 1.47 (H-13 & H-17, *m*, 2H); 1.62 (H-3, *m*, 2H); 2.05 (H-8, *m*, 2H); 2.21 (H-11, *m*, 2H); 2.31 (H-2, *t*, 2H); 3.62 (H-12, *m*, 1H); 3.67 (OCH_3_, *s*, 3H); 5.42 (H-10, *m*, 1H); 5.55 (H-9, *m*, 1H). ^1^H and ^13^C-NMR spectral data of compound **2** were in good agreement with ricinoleic acid methyl ester spectral data reported in the National Center for Biotechnology Information (NCBI) [56].

### 4.8. Caenorhabditis Elegans Assay

Anthelmintic assay was carried out on the model nematode *C. elegans* using the method developed by Thomsen et al. [57]. The nematodes were cultured on NGM Petri plates using uracil auxotroph *Escherichia coli* strain OP50 as a food source. After 4 days of cultivation, the nematodes were transferred from the Petri plate to a 15 mL falcon tube by rinsing each plate twice with 2 mL of M9 buffer. The worm suspension was then centrifuged for 1 min at 800 rpm. After removal of the supernatant, the nematodes were washed again with 2 mL of M9 buffer under the same conditions, and, depending on the number of animals, re-suspension was carried out in 2 to 8 mL of M9 buffer. To this suspension, 10 µL penicillin–streptomycin solution (10 mg/mL) was added. The assay was performed in 384-well plates after adjusting the worm number to 20–30 per 20 µL. The outer wells were filled with 40 µL of water to minimize evaporation prior to incubating 20 µL of worm suspension with 20 µL of test solution. The number of living and dead animals in each well was then counted using an inverted cell culture microscope (Olympus CKX41, Olympus Life Science, Waltham, MA, USA) after 30 min. Dimethyl sulfoxide (DMSO 2%) was used as a negative control, while ivermectin (10 µg/mL) was used as a positive control. All the assays were done in triplicate.

### 4.9. Molecular Docking Studies

#### 4.9.1. Structure Validation of Homology Modeled Protein

Ricinoleic acid was subjected to molecular docking using the Schrödinger suite 2023 version 1 (LLC; New York, NY, USA, 2022) program [58]. The target proteins for this docking analysis included SDH, G6PD and TBB2C protein structures from *C. elegans*. These proteins are commonly recognized as valid targets for various anthelmintic activities [59]. Due to the absence of crystallographic structures for SDH, G6PD and TBB2C in the Protein Data Bank (PDB) (accessed on 5 January 2024 https://www.rcsb.org), homology modeling was used to build a 3D model of the target proteins. The amino acid sequence of SDH, G6PD and TBB2C protein structures form *C. elegans* was retrieved from Uniprot database UniProtKB (Universal Protein Resource Knowledgebase) webserver, http://www.uniprot.org with primary accession code: Q09508, Q27464 and P52275, respectively [60]. The sequence was then uploaded on Basic Local Alignment Search Tool (BLAST) using the SWISS-MODEL online workspace (https://www.swissmodel.expasy.org) (SIB, Lausanne, Switzerland) program [61] to construct homology model.

The quality of the modeled 3D protein structures was assessed using the Qualitative Model Energy Analysis (QMEAN) and Global Model Quality Estimation (GMQE) scores as well as Ramachandran Plots. The QMEAN score of below − 4.0 depicts low quality of the predicted structure, while the GMQE score ranges between 0 and 1, and a higher score corresponds to higher reliability [62,63]. Finally, the models with the best features were selected and ready for protein preparation procedures.

#### 4.9.2. Preparation of the Target Proteins

Following the validation stage, the proteins underwent preparation using the Schrödinger suite 2023 version 1 program [58]. This involved adding hydrogens, addressing issues such as missing side chains, assigning correct bond orders, and adjusting the ionization and tautomeric states through the use of the Epik tool. All the water molecules beyond 5 Å were deleted. Optimization and subsequent minimization were carried out on all the atoms using OPLS3 force fields, with the convergence of heavy atoms to RMSD set to 0.3 Å (default).

#### 4.9.3. Ligand Preparation

ChemDraw Ultra (2019) was used to draw the structure of ricinoleic acid in MDL SDfile format. This compound was converted into an energy-minimized 3D structure using the LigPrep wizard of Schrödinger maestro (v13.5). With the assistance of Epik, the possible ionization state was generated at target pH 7.0 ± 2.0 for accurate tautomer enumeration and to know the protonation state in biological status [64]. The force field was set to OPLS4 to determine the stereoisomers of ricinoleic acid with specified chirality.

#### 4.9.4. Prediction for Active Protein Site

In the docking experiment, the active sites in the modeled protein structure were predicted by using the Receptor Grid Generation tool in Maestro [58]. A grid box containing the x, y, and z coordinates at the binding site residues of native co-enzymes and substrates was created for docking.

#### 4.9.5. Ligand Docking

After completing the necessary preliminary steps, docking was carried out using the homology-modeled and prepared 3D molecular structures of SDH, G6PD, and TBB2C from *C. elegans*. This process utilized a standard precision scoring function (SP), followed by the extra-precision (XP) scoring function for enhanced accuracy. The definitive score, expressed as the glide score, was determined using energy-minimized postures. Subsequently, the scores for ricinoleic acid and the standard drugs in each activity were recorded.

#### 4.9.6. ADME Descriptors Analysis

ADME (i.e., absorption, distribution, metabolism and excretion) plays a crucial role in assessing the drug-like properties of a compound, and these properties are evaluated based on Lipinski’s rule of five [35,36]. In this study, the QikProp module of Schrödinger Maestro (v13.5) was employed to determine the ADME properties of ricinoleic acid [35]. The accessibility of a compound throughout the body, as determined by Lipinski’s rule of five [35,36], is reflected in its ADME properties. Lipinski’s rule of five sets standard parameters, including a molecular weight, a hydrogen bond donor count, a hydrogen bond acceptor count, lipophilicity, and a molar refractivity.

### 4.10. Data Analysis

Data is presented in terms of the average percentage of dead worms to the total number of nematodes. The results were analyzed by using one-way ANOVA (Tukey’s multiple comparison test) using the SPSS program version 25. A confidence interval of 95% was used, and *p* < 0.05 was considered significant.

## 5. Conclusions

The present study demonstrates that castor oil, ricinoleic acid, and its methyl ester possess genuine anthelmintic activity against *C. elegans*. Molecular docking was conducted on SDH, G6PD, and TBB2C proteins, as these proteins were identified as potential targets for anthelmintic drugs. This study sheds light on predicting possible interaction modes and binding affinities of ricinoleic acid with homology-modeled SDH, G6PD, and TBB2C. Among these models, ricinoleic acid exhibited the highest docking value with SDH, comparable with that of the standard drug mebendazole, which is used to treat intestinal worm infections such as pinworm, roundworm, and hookworm. However, to validate the protein target, experimental characterization is necessary. The present findings also confirm that ricinoleic acid is fully or partially responsible for the anthelmintic activity of *R. communis* seeds.

## Figures and Tables

**Table 1:** Validation of parameters used for the predicted protein model through homology modeling.

Enzymes	GMQE Score	QMEAN Score	Sequence Identity Percentage	Ramachandran Plot
SDH	0.86	−0.89	73.62%	96.39% favored
G6PD	0.86	−0.75	60.53%	94.61% favored
TBB2C	0.82	−1.27	89.16%	96.50% favored

**Table 2:** Molecular docking scores of ricinoleic acid against succinate dehydrogenase (SDH), glucose-6-phosphate 1-dehydrogenase (G6PD), and tubulin beta-2 chain enzymes (TBB2C).

No	Enzyme	Docking Score (kcal/mol)		Glide Score (kcal/mol)		Glide Model (kcal/mol)	
		Ricinoleic Acid	Mebendazole	Ricinoleic Acid	Mebendazole	Ricinoleic Acid	Mebendazole
1	SDH	−5.408	−6.243	−5.413	−6.558	−71.326	−66.032
2	G6PD	−3.758	−3.814	−3.763	−4.130	−31.923	−41.351
3	TBB2C	−1.444	−4.756	−1.449	−5.072	−35.155	−50.874

**Table 3:** Molecular docking of ricinoleic acid *.

No	Enzyme	H–Bonds AA	Non–H–Bonds AA
1	SDH	Arg433, Ala436, Ala236	Thr72, Leu442, Ala50, Gly49, Ala53, Thr237, Lys73, Leu74, Thr81, Ser438, Leu439, Glu422, Ala436, Gly85, Hie80
2	G6PD	Gly403, Arg252	Lys49, Lys211, 297, Lys177, Glu176, Ala146, Leu147, Pro148, Leu45, Tyr445
3	TBB2C	Cys12, Asp177	Leu225, Ala228, Trp231, Met213, Val214, Gln215, Asn216, Leu217, Met218, Val219, Arg221, Thr221, Phe222, Lys281

Note: SDH: succinate dehydrogenase; G6PD: glucose-6-phosphate 1-dehydrogenase; TBB2C: tubulin beta-2 chain. * See Figure 6, Figure 7 and Figure 8 for graphical representations.

**Table 4:** ADME properties of ricinoleic acid.

Descriptor	Predicted Values	Recommended Values [36]
Molecular weight (mol MW)	298.465 g/mol	130.0–725.0
Hydrogen bond donor (donorHB)	2	0.0–6.0
Hydrogen bond acceptor (acceptHB)	3.7	2.0–20.0
High lipophilicity (QPlogP_o_/W)	4.801	−2.0–6.5
Molar refractivity (QPpolrz)	32.945	13–70.0

## Data Availability

The data that support the findings of this study are available in the Supplementary Materials of this article. If there are more requirements, the data are available from the corresponding author upon reasonable request.

## Supplementary Materials

The following supporting information can be downloaded at https://www.mdpi.com/article/10.3390/ijms26041636/s1.
