# Supplementary material for: Anthelmintic Potential and In Silico Studies of Ricinoleic Acid from the Seed Oil of Ricinus communis L
Source: Int J Mol Sci. 2025 Feb 14;26(4):1636. doi: 10.3390/ijms26041636 (PMC11855838; doi:10.3390/ijms26041636)
Supplement: Supplementary file 1 [file ijms-26-01636-s001.zip › ijms-3391950-supplementary.pdf]

Supporting Material

Anthelmintic Potential and *In Silico* Studies of Ricinoleic Acid from the Seed Oil of *Ricinus communis* L.

By Temesgen Berhanu[1,2], Eyael Tewelde[1], Mariamawit Y. Yeshak[1], Daniel Bisrat[1] and Kaleab Asres[1*]

[1] Department of Pharmaceutical Chemistry and Pharmacognosy, School of Pharmacy, College of Health Sciences, Addis Ababa University, P.O. Box 1176, Addis Ababa, Ethiopia;

[2] Department of Pharmacognosy, School of Pharmacy, Dilla University, P.O. Box 419, Dilla, Ethiopia

[*]Corresponding author: Kaleab Asres Tel.: +251911404249; E-mail: kaleab.asres@aau.edu.et

Temesgen Berhanu: E-mail: tederastame@gmail.com

Tel.: +251926421378

Eyael Tewelde: E-mail: eyaeltd@gmail.com

Tel.: +251912387457

Daniel Bisrat: E-mail: daniel.bisrat@aau.edu.et

Tel.: +251 987100037

Kaleab Asres: E-mail: kaleab.asres@aau.edu.et

Tel.: +251911404249

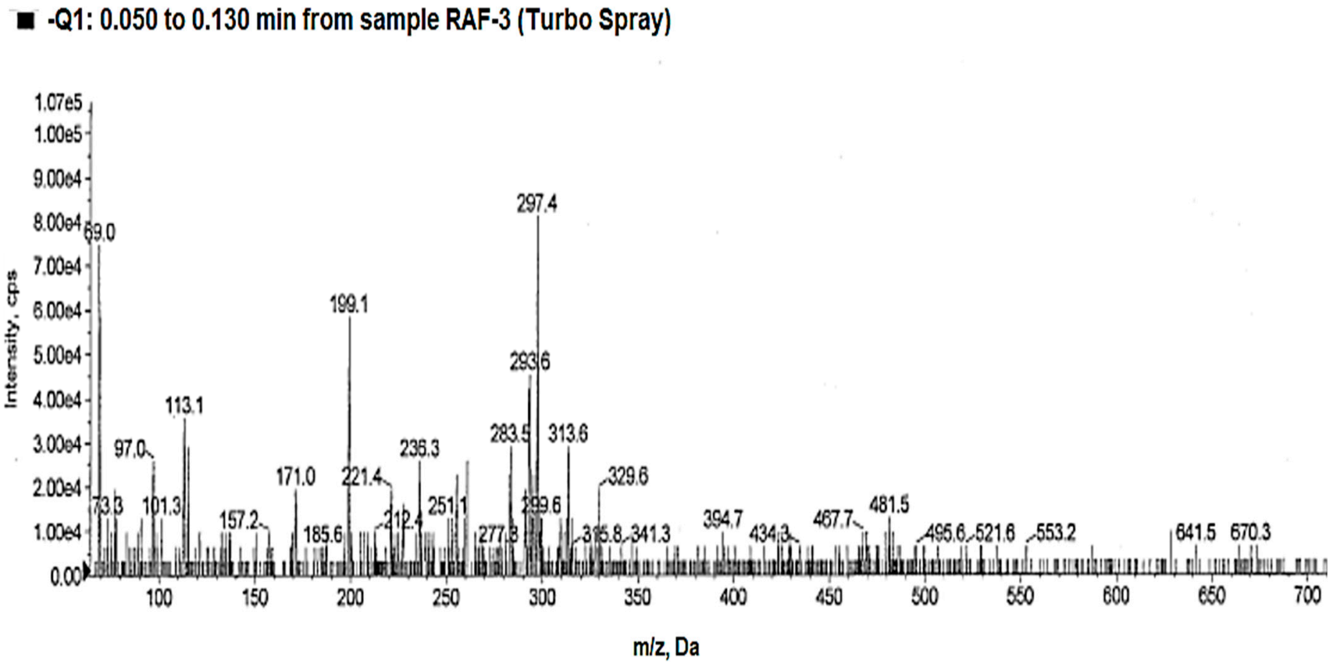

Figure S1. Negative-mode ESI-mass spectrum of ricinoleic acid (**1**)

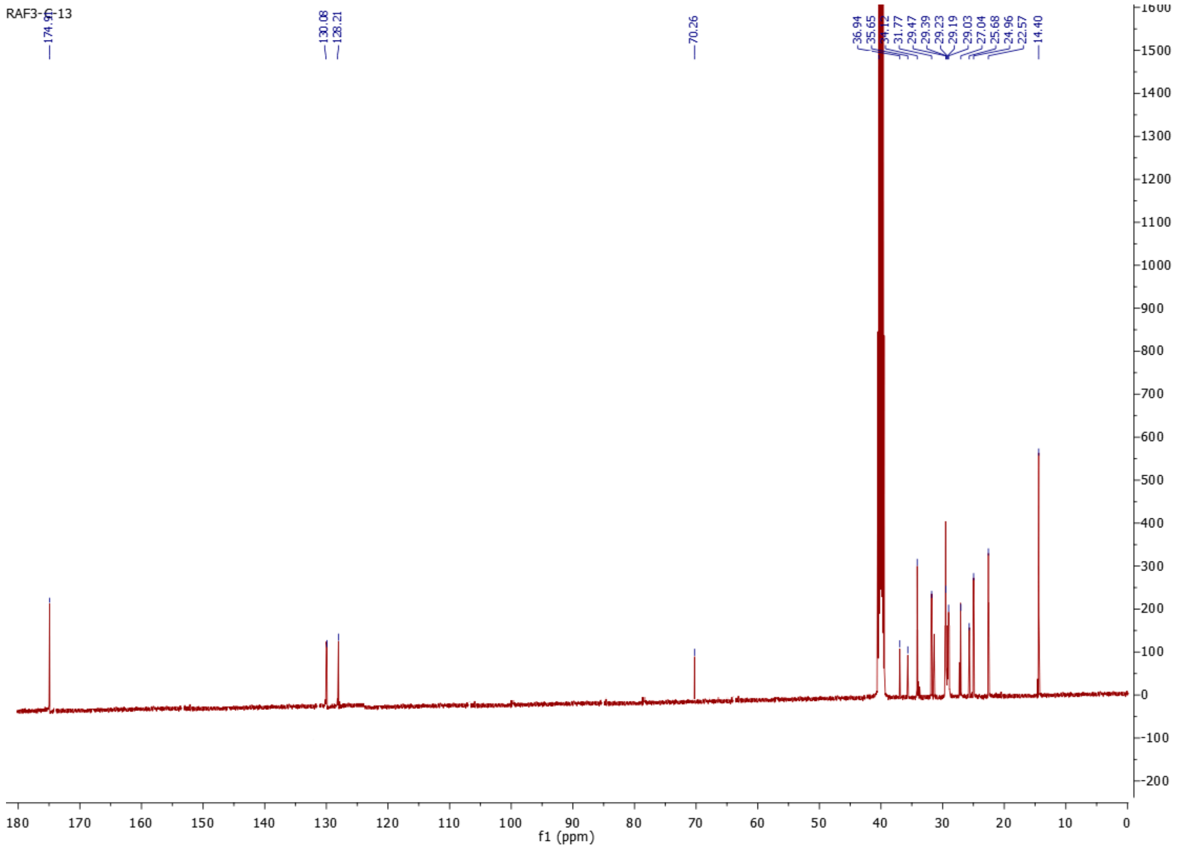

Figure S2. ^{13}C-NMR spectrum of ricinoleic acid (**1**) in deuterated DMSO.

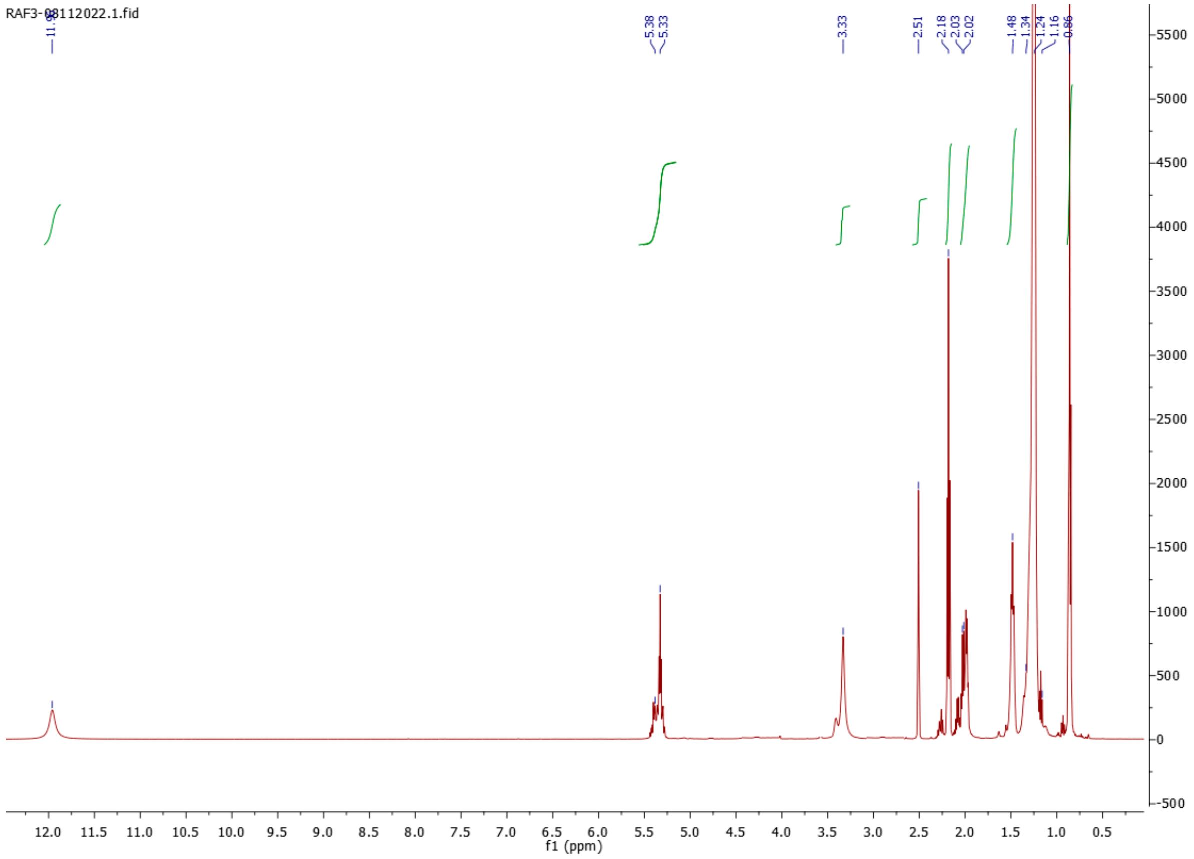

Figure S3. ^{1}H-NMR spectrum of ricinoleic acid (**1**) in deuterated DMSO

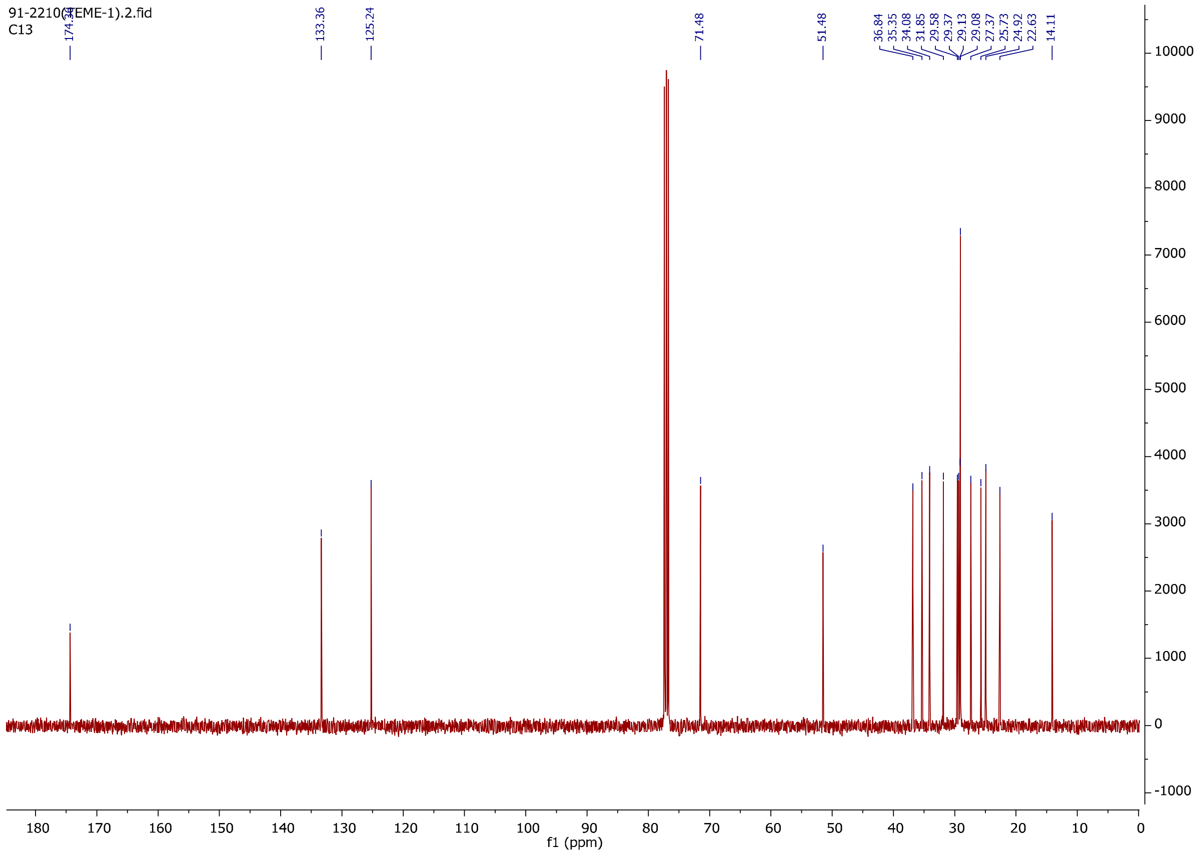

Figure S4. ^{13}C-NMR spectrum of methyl ricinoleate (**2**) in deuterated $CDCl_3$.

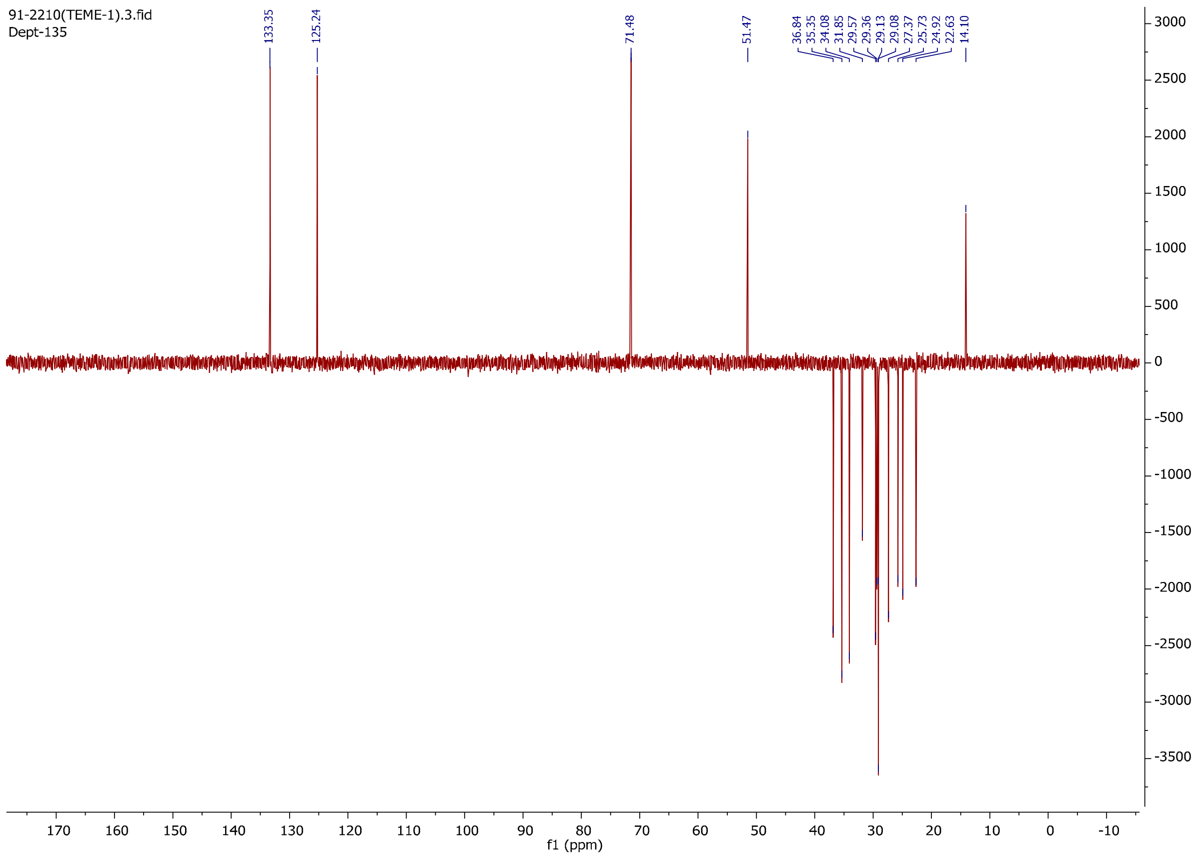

Figure S5. DEPT-135 spectrum of methyl ricinoleate (**2**) in deuterated $CDCl_3$

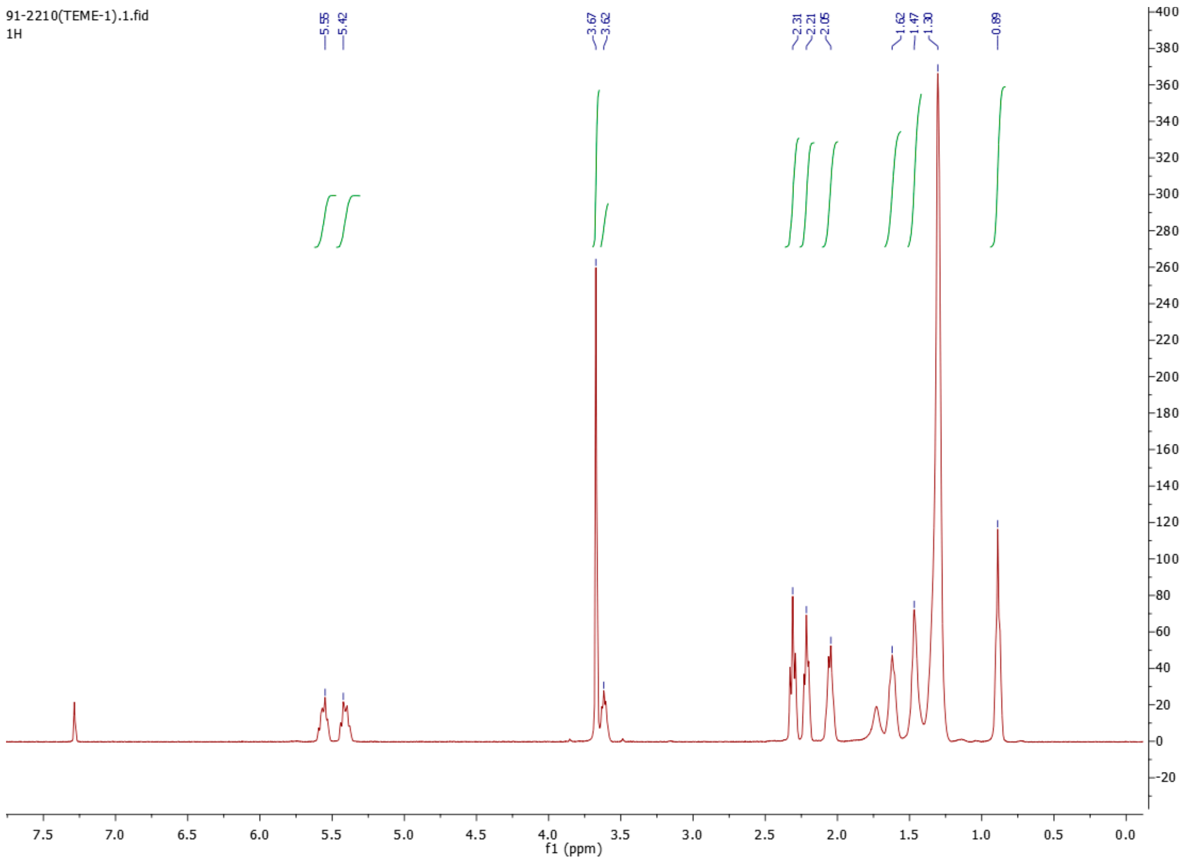

Figure S6. ^{1}H-NMR spectrum of methyl ricinoleate (**2**) in deuterated $CDCl_3$
